# Characterization of Bacteria and Inducible Phages in an Intensive Care Unit

**DOI:** 10.3390/jcm8091433

**Published:** 2019-09-10

**Authors:** Cátia Pacífico, Miriam Hilbert, Dmitrij Sofka, Nora Dinhopl, Ildiko-Julia Pap, Christoph Aspöck, Friederike Hilbert

**Affiliations:** 1 Department of Farm Animals and Veterinary Public Health, University of Veterinary Medicine, 1210 Vienna, Austria; 2 Karl Landsteiner University of Health Sciences, 3500 Krems an der Donau, Austria; 3 Department of Pathobiology, University of Veterinary Medicine, 1210 Vienna, Austria; 4 Institute of Hygiene and Microbiology, University Clinic St. Pölten, 3100 St. Pölten, Austria

**Keywords:** bacteriophages, *Staphylococcus aureus*, *Escherichia coli*, intensive care unit, prophage induction

## Abstract

Intensive care units (ICUs) are critical locations for the transmission of pathogenic and opportunistic microorganisms. Bacteria may develop a synergistic relationship with bacteriophages and more effectively resist various stresses, enabling them to persist despite disinfection and antimicrobial treatment. We collected 77 environmental samples from the surroundings of 12 patients with infection/colonizations by *Escherichia coli*, *Staphylococcus aureus* or *Klebsiella* spp. in an ICU in Austria. Surface swabs were tested for lytic phages and bacterial isolates for mitomycin C-inducible prophages. No lytic bacteriophages were detected, but *S. aureus* was isolated from the surroundings of all patients. About 85% of the colonies isolated from surface samples were resistant to antimicrobials, with 94% of them multidrug resistant. Two inducible temperate bacteriophages—myovirus vB_EcoM_P5 and siphovirus vB_SauS_P9—were recovered from two clinical isolates. Staphylococci phage vB_SauS_P9 lysed *S. aureus* isolates from the surface swabs collected from the surroundings of three patients. No transductants were obtained on propagation in phage-sensitive antimicrobial-resistant isolates. The two phages were sensitive to 0.25% (v/v) of the disinfectant TPH Protect, which eliminated viable phages after 15 min. Coliphage vB_EcoM_P5 was inactivated at 70 °C and staphylococci phage vB_SauS_P9 at 60 °C after 60 min.

## 1. Introduction

The intensive care unit (ICU) has been defined as an “epicenter” of nosocomial infections. Most of these pathogens are involved in the development, propagation, and amplification of antimicrobial resistance [1,2]. Infections caused by bacteria resistant to antimicrobials are correlated with age, severity of disease, general debility, length of hospitalization, use of invasive devices, and recurrent antimicrobial treatment [1,3,4]. Medical equipment, environment, patients, and hospital staff may be contaminated by pathogenic or opportunistic bacteria [5], such as methicillin-resistant *Staphylococcus aureus* (MRSA) and carbapenem-resistant *Enterobacteriaceae* [1,6]. Hospital hygiene policies mandate personal hygiene, active surveillance of asymptomatic colonization [7], decolonization strategies [8], and in severe cases, single-room isolation of MRSA-harboring patients [9]. Patients with *Enterobacteriaceae* may be asymptomatic, as these bacteria can effectively colonize the human gut and have a natural role as commensal organisms. Decontamination, thus, relies on an appropriate disinfection regime to prevent the dissemination of pathogens and improve the outcome of treatment [8,10].

Data from the European Centre for Disease Prevention and Control show that healthcare-associated infections in hospitals cause more deaths in Europe than any other infectious disease [11]. Bacteriophages—bacterial viruses—have been isolated from the bacteria of septicemic patients and free phage particles have been detected in patients’ blood at the onset of sepsis [12]. However, there have been a few studies on the ecological aspect of bacteriophages in clinical bacterial infections [12,13,14] or colonizations [15].

Phages are the most abundant members of the human virome, present in every community examined [16,17,18]. Their wide distribution in the environment impacts both viral diversification and the bacterial host, shaping microbial communities towards an expanded functional diversity of the ecosystems. Lytic phages kill their bacterial host cell through lysis, while temperate bacteriophages (or lysogenic phages) either integrate in the bacterial genome (forming a so-called prophage) or exist as a plasmid in the bacterial cytoplasm. In the event of environmental stress, functional prophages can be excised and enter a lytic cycle [16,19,20]. The existence of prophages in the bacterial genome acts as a supplementary gene pool of horizontally transferred genes that confers higher fitness to the bacteria, thanks e.g., to the presence of virulence genes, antimicrobial resistance genes and/or survival factors [21,22,23,24]. The resulting lysogenic bacteria can cause human diseases, such as Shiga-toxin induced hemolytic uremic syndrome due to phage-encoded endotoxins [25], staphylokinase, chemotaxis inhibitors, staphylococcal complement inhibitor, or factors associated with biofilm formation [26].

The development of high-throughput sequencing technologies has enabled the complete characterization of microbiomes, including not only bacteria [27] but also viruses [28], and attention is now turning to how the microorganisms interact with the environment and with one another. Nevertheless, previous work on colonization of the ICU by commensal and pathogenic bacteria [28,29,30,31] has not considered the role of bacteriophages. We report an investigation of important ICU pathogens and their phages in the surroundings of patients colonized/infected with *S. aureus*, *K. oxytoca*, *K. pneumoniae*, and *E. coli*. Two lysogenic isolates retrieved from patients were able to release prophages upon stimulation with mitomycin C. The bacteriophages were tested for the ability to mediate the transfer of antimicrobial resistance genes from resistant bacteria identified in the vicinity of the patients to sensitive host strains.

## 2. Materials and Methods

### 2.1. Intensive Care Unit

Between February 2017 and March 2018, a total of 77 surface samples were collected from the surroundings of twelve patients (P1 to P12) in an ICU of a hospital in the region of Lower Austria. The floor of the ICU is cleaned and disinfected once a day, and after every contamination. The sanitary installations (toilets, sinks, and faucets) are cleaned once a day with a disinfecting sanitary cleaner. Surfaces far from patients and with frequent hand contact are disinfected once a day with a regular quaternary ammonium-based disinfectant (TPH Protect 0.5% v/v). Surfaces in close contact with patients are disinfected at least twice a day and always after contamination, while devices are disinfected after each patient, at least twice a day and after contamination. Blankets and pillows are washed after contamination. In the event of *Clostridia difficile* colonization/infection, surfaces and floors are continuously disinfected with a sporocidal disinfectant (peracetic acid), and after regular hand disinfection, the staff is instructed to wash their hands with water and soap. The regular hand disinfectant is effective against Norovirus, but in the event of an infection with the virus, the surfaces are disinfected more frequently with the regular quaternary ammonium-based disinfectant (four times a day) and there is a terminal disinfection with peracetic acid.

The clinical isolates from patients P1 to P10 were collected (no clinical isolates were available from patients P11 and P12), together with relevant patient information. Additionally, 20 *E. coli* and 17 *S. aureus* isolates from stationary patients and outpatients in the hospital during the sampling period were included to test the bacteriophage host range (see Table 3). The study was performed in accordance with the Declaration of Helsinki and the institutional rules for Good Scientific Practice. The samples were anonymized and no confidential data from the patients was collected. Samples were collected from defined sites (pillow, breathing hose pole, blanket, bed rail, infusion bag, emergency/control button, dietary pump, side table, mattress topper, sheets, switch panel, feeding tube, and respiratory tube) using gel swabs (Transsystem™, COPAN, Brescia, Italy), liquid swabs (eSwab™, COPAN, Brescia, Italy), or surface wipes (Polywipe®, MWE, Wiltshire, England) (Table 1). The samples were diluted in 1–3 mL of sterile 0.9% NaCl (Merck, Darmstadt, Germany) and vortexed. The rinsed saline solution was used for the isolation of phages and bacteria. Bacterial cell counts were calculated per swab, or per wipe. The samples were collected prior to disinfection of the patient’s surroundings and sent immediately for laboratory analysis.

### 2.2. Isolation of Bacteria from Environmental Samples

Isolates from *E. coli* were quantified on Coli-ID agar plates (bio-Mérieux, Marcy l’Etoile, France) and *S. aureus* on Aureus Agar Base HiCrome (Sigma-Aldrich, MO, USA) by plating 50 µL of the rinsing solution. For the detection of the presumed resistant bacteria, five colonies were collected from each environmental sample and incubated for 24 h at 37 °C on Mueller-Hinton agar (Oxoid Ltd., Basingstoke, UK) containing appropriate antimicrobials. *E. coli* were tested for resistance towards ampicillin at 35 µg/mL, tetracycline at 20 µg/mL, kanamycin at 30 µg/mL, and chloramphenicol at 35 µg/mL, while *S. aureus* were tested for penicillin-G at 0.5 µg/mL, erythromycin at 4 µg/mL, clindamycin at 1 µg/mL, and tetracycline at 20 µg/mL (Sigma-Aldrich, St. Louis, MO, USA). The choice of antibiotics was based on their widespread use in veterinary and human medicine [32]. Disc diffusion was performed using the above-mentioned antimicrobials, according to the hospital’s laboratory routine following guidelines of the European Committee on Antimicrobial Susceptibility Testing (EUCAST) [33] (see below). Resistant bacteria were re-cultured on AB-plates with appropriate antibiotics. The isolated bacterial colonies were stored in Modified Scholtens’ Broth (MSB) or 2× Yeast Tryptone (YT) media with the addition of 20% (w/v) glycerol (Sigma-Aldrich, St. Louis, MO, USA) at −80 °C.

### 2.3. Lytic Bacteriophage Detection

All 77 samples were tested for lytic bacteriophages infecting either *E. coli* or *S. aureus* according to ISO 10706-2:2000 [34], as described in [32,35]. Both *E. coli* DSM 12242 and *S. aureus Sa9* [36] were used as indicator host strains for bacteriophage detection. An overnight culture of the indicator bacterium was used to inoculate a new broth and grown to an optical density of 0.4 at 600 nm. One milliliter of the fresh bacterial culture and 1 milliliter of rinsed saline solution were added to 3 mL of preheated MSB, supplemented with 10 mM CaCl_2_. The mixture was vortexed and overlaid on Modified Scholtens´ Agar (MSA) at room temperature. The solidified plates were incubated overnight at 37 °C.

### 2.4. Clinical Isolates from Patients

Bacterial isolates and antibiograms were obtained for patients P1-P10. We did not evaluate whether patients were colonized or infected and isolates were not necessarily the cause of hospitalization (Table 2). Disc diffusion was performed according to the hospital’s laboratory routine, following EUCAST guidelines [36]. For *Enterobacteriaceae,* we tested ampicillin (AM10), cefpodoxime (CPD10), cefuroxime (CXM30), cefotaxime (CTX5), ampicillin/sulbactam (SAM20), cefepim (FEP30), gentamicin (CN10), imipenem (IPM10), ceftazidime (CAZ10), meropenem (MEM10,) cefoxitin (FOX30), ciprofloxacin (CIP5), amikacin (AK30), and trimethropim (TMP5). *S. aureus* was tested for rifampicin (RA5), linezolid (LNZ10), mupirocin (MUP200), trimethropim (TMP5), fosfomycin (FF200), fusidic acid (FA10), minocyclin (MI30), gentamicin (CN10), erythromycin (E15), clindamycin (DA2), tetracycline (TE30), moxifloxacin (MXF5), cefoxitin (FOX30), penicillin G (P1), and tigecycline (TGC15).

### 2.5. Prophage Induction

The ten bacterial isolates from each patient and a subset of bacteria (*n* = 73) collected from the environmental samples were tested for prophage induction using 0.5 mg/mL mitomycin C (AppliChem GmbH, Darmstadt, Germany). Briefly, 40 mL of MSB media was inoculated with fresh bacteria and grown until an optical density of 0.4 at 600 nm. At this point, 2 to 5 µg/mL mitomycin C and 10 mM CaCl_2_ were added and incubated for 2 h. After incubation, the cells were supplemented with 10 mg/mL lysozyme (Sigma-Aldrich, St. Louis, MO, USA) to stimulate prophage release. The suspension was re-incubated and the supernatant collected after 24 h. The supernatants were centrifuged for 5 min at 8000 g and plated following the soft-agar overlay method (as outlined above), using the native host (patient isolate, environmental isolate) and the indicator bacteria (*S. aureus* Sa9 or *E. coli* DSM 12242). A control without any inducing agent was included to test for spontaneous phage release.

### 2.6. Bacteriophage Purification, Propagation, and Lysate Preparation

Phage lysates were prepared according to Groisman [37]. Four individual plaques from each sample were retrieved from the soft agar layer and each suspended in 1 mL media. The solution was incubated at 37 °C for 30 min, further filtered through a 0.2 µm-pore filter and stored at 4 °C. Bacteriophage suspensions were propagated by re-infection of the indicator host in triplicate using the purified lysate and the soft agar overlay method. After overnight incubation at 37 °C, the soft agar was shredded and 3 mL of media were added. The overlay was collected and centrifuged at 8000 g for 5 min. The clear supernatant was filtered through a 0.2 µm-pore filter and kept at 4 °C. Tenfold serial dilutions (10^−1^ to 10^−6^) were prepared and 10 µL of the diluted lysate was plated with the indicator bacteria. After overnight incubation at 37 °C, the plaques were counted and the titer expressed as PFU/mL.

### 2.7. Transmission Electron Microscopy (TEM)

To prepare the phage for transmission electron microscopy (TEM), a droplet of the purified phage suspension (10^7^–10^8^ PFU/mL) was deposited on a copper grid (Science Services, Munich, Germany) with carbon-coated Formvar film for 10 min at room temperature and stained with 4% aqueous phosphotungstic acid (Merck, Darmstadt, Germany) at pH 7. After air drying overnight, the sample was observed with the Zeiss TEM 900 electron microscope (Carl Zeiss, Oberkochen, Germany) operated at 50 kV, using Image SP software and a CCD camera (TRS, Tröndle Restlichtverstärkersysteme, Moorenweis, Germany).

### 2.8. Host Range Analysis

Phages vB_EcoM_P5 and vB_SauS_P9 were tested for the ability to lyse the patient isolates collected during this study, bacterial collection strains, and clinical isolates from stationary patients and outpatients (see Table 3). Host range was determined by spot assay. A volume of 0.2 mL of stationary phase cell suspension was mixed with 3 mL of Molten-soft agar supplemented with 10 mM CaCl_2_, poured into a MSB or 2×YT agar plate. After solidification, 0.02 mL of a 10^7^ PFU/mL phage suspension was spotted on the overlay and incubated at 37 °C overnight. Phage vB_SauS_P9 was also tested in the *S. aureus* colonies isolated from the environmental samples taken during the study (*n* = 150).

### 2.9. Determination of Efficiency of Plating (EOP)

Bacterial lysis was confirmed by calculating the efficiency of plating (EOP) regarding the indicator host *S. aureus* Sa9 or *E. coli* DSM 12242 (EOP = 1.0). A new bacterial host culture was prepared as above. One mL of host culture and 0.01 mL of a 10^7^ PFU/mL phage suspension were added to 3 mL of preheated MSB supplemented with 10 mM CaCl_2_. The mixture was vortexed, overlaid on MSA at room temperature, and incubated overnight at 37 °C. EOP was defined as the ratio between PFU/mL on the sensitive bacteria and the PFU/mL on the indicator strain. The bacteria were graded by their level of sensitivity as “high” (EOP ≥ 0.5), “medium” (0.1 ≤ EOP < 0.5), “low” (0.001 ≤ EOP < 0.1), and “inefficient” (EOP ≤ 0.001).

### 2.10. Bacteriophage Transduction

Bacteriophage transduction of vB_EcoM_P5 was performed using E. coli DSM 12242 as a host. Briefly, 100 µL of an overnight culture was mixed with 10 µL of phage lysate and incubated at 37 °C for 30 min to permit phage absorption. Two milliliters of MSB was added and the mixture was incubated at 37 °C for 1 to 2 h with shaking. The tubes were centrifuged at 8000 g for 3 min. The supernatant was discarded, and the pellet suspended in 150 µL MSB and plated on Mueller-Hinton agar plates containing ampicillin at 35 µg/mL. Incubation was performed for approximately 48 h at 37 °C. As bacteriophage vB_SauS_P9 was derived from an AB-sensitive S. aureus, it was first propagated in two different antibiotic-resistant isolates from the environment of P5, with resistance against penicillin G, erythromycin, and clindamycin to possible resistance genes to be transduced. The new lysates of vB_SauS_P9 were used for transduction, as described above. The suspended pellet was plated on Mueller-Hinton agar containing 0.5 µg/mL penicillin-G, 4 µg/mL erythromycin, and 1 µg/mL clindamycin.

### 2.11. Disinfectant, Ethanol and Thermal Stability Tests

Phage stability was determined in the presence of common hospital disinfectants, TPH Protect and Hexaquart^®^ plus, at concentrations of 0.25% and 0.5%. Phage preparations of 107 PFU/mL were incubated at room temperature and phage titer was determined at 0, 15, 30 and 60 min by serial dilutions tested on the indicator strain by means of the soft agar overlay method. The phage particles were counted after overnight incubation at 37 °C. Assays were performed in duplicate. Survival to 70% ethanol and temperature stability (25, 37, 45, 44, 60, 65 and 70 °C) was examined after 60 min incubation, as described elsewhere [38]. None of the conditions we tested affected the bacterial host strain, and phage infection was not impeded.

## 3. Results

### 3.1. Bacterial and Viral Contamination

**Figure 1:**
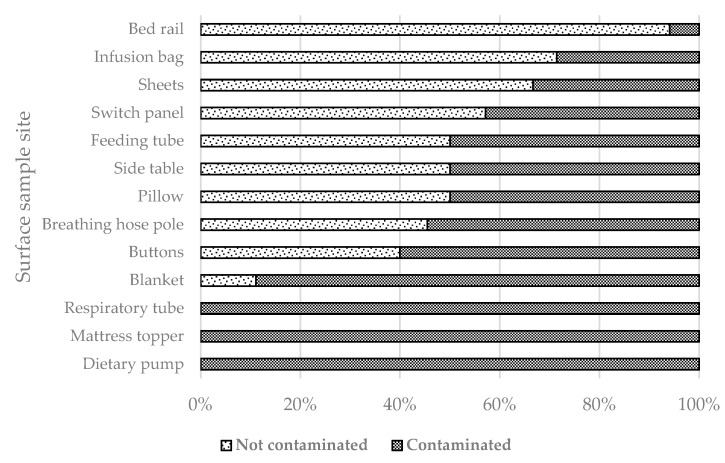
*Staphylococcus aureus* contamination of the environmental samples, according to the percentage of contaminated versus non-contaminated sites.

*Staphylococcus aureus* was detected in the vicinity of all 12 patients, independent of the bacterial agent of infection/colonization. A total of 36 samples (circa 47%) were found to be positive. Most often, samples taken from blankets were positive for *S. aureus* (88.9%), followed by emergency/control buttons (60.0%) and breathing hose poles (54.5%). Other settings, such as respiratory tubes, mattress toppers, and dietary pumps were contaminated in every single case (*n* = 3, 2 and 1, respectively) (Figure 1). Higher levels of contamination were found on sheets, pillows, buttons, and blankets. *E. coli* was not isolated in any of the surface samples.

**Figure 2:**
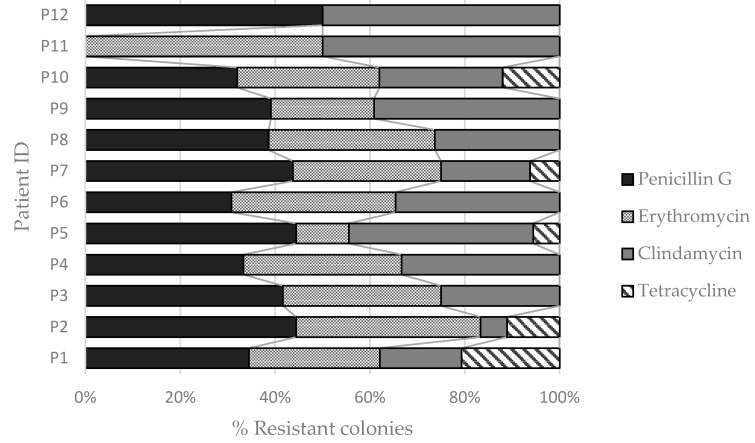
Percentage of environmental bacteria resistant to penicillin G, erythromycin, clindamycin, and tetracycline, found in the surroundings of each patient.

The majority of the colonies of *S. aureus* isolated from the environmental samples were resistant to at least one of the four antimicrobials tested (*n* = 128). Only 22 isolates were sensitive to all four antimicrobials. Of the resistant ones, 120 were multidrug resistant, of which 111 were resistant to two or three of the antibiotics tested and 9 were resistant to all four. Only 8 were resistant to only one antibiotic. Most *S. aureus* isolates were resistant to penicillin G (123 of 150 isolates), followed by erythromycin (100 out of 150), clindamycin (98 of 150), and tetracycline (16 of 150) (Figure 2).

Neither lytic bacteriophages lysing the indicator strains *E. coli* DSM 12242 nor *S. aureus* Sa9 were detected in any of the samples.

### 3.2. Temperate Phages

**Figure 3:**
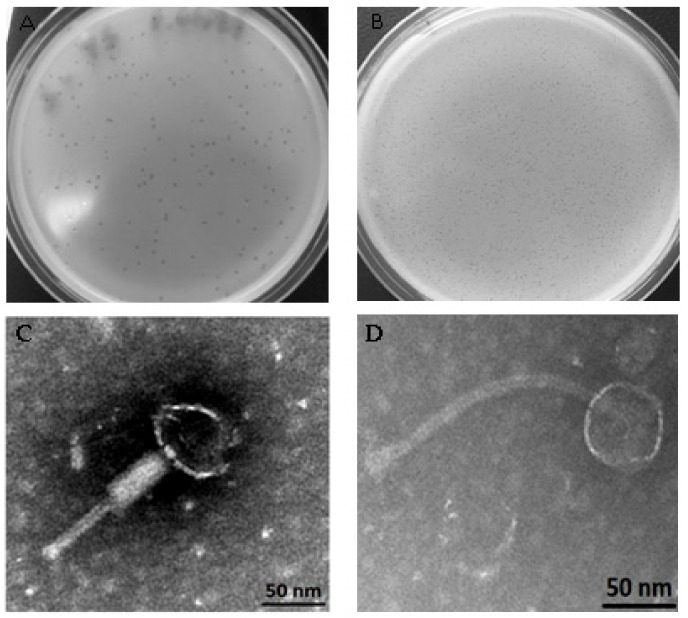
Plaque and virion morphologies of coliphage vB_EcoM_P5 (**A**,**C**) and staphylococci phage vB_SauS_P9 (**B**,**D**).

The presence of mitomycin C-inducible prophages was investigated in the 10 bacterial isolates collected (P1 to P10) and in a subset of 73 *S. aureus* isolates from the environmental samples. Of these, two of the patients’ bacterial isolates contained mitomycin-inducible prophages, corresponding to an *E. coli* and an *S. aureus* isolate (P5 and P9, respectively). None of the isolates from environmental samples released phage particles spontaneously or upon mitomycin C induction. The phages were purified by single-plaque propagation (Figure 3A,B) and named vB_EcoM_P5 and vB_SauS_P9. Phage vB_EcoM_P5 formed transparent, small, round plaques, approximately 0.3 mm in diameter on the lawn of *E. coli* DSM 12242, while phage vB_SauS_P9 formed rather small plaques of 0.1 mm under the conditions tested. Transmission electron micrographs of vB_EcoM_P5 (Figure 3C) and vB_SauS_P9 (Figure 3D) show phages with an icosahedral head of approximately 50–52 nm. The coliphage has a tail of about 140 nm, while the staphylococci phage has a longer tail of approximately 200 nm. Based on virion morphology, vB_EcoM_P5 was classified as a *Myoviridae* family phage and vB_SauS_P9 as a *Siphoviridae* family phage (Figure 3C,D) [39].

### 3.3. Lytic Spectrum

The two phages were tested for the ability to lyse environmental bacteria and other clinical isolates collected during the study. Staphylococci-infecting phage vB_SauS_P9 lysed 45 of 150 environmental isolates collected from the patients’ surroundings. The colonies were collected in the surroundings of P5, P7 and P8, all representing *Enterobacteriaceae* infections/colonizations. A higher sensitivity was found in the bacteria collected from the surroundings of patient 5 (0.1 ≤ EOP < 1.0). The remaining surface isolates were categorized as being low and inefficient producers, according to the calculated EOP. Of the seventeen *S. aureus* clinical isolates tested, only one was sensitive to phage vB_SauS_P9 (EOP = 0.02). *S. aureus* isolate 24 was previously collected in the hospital. None of the *S. aureus* isolates (*n* = 3) from the 10 patients nor the culture collection strains (*n* = 2) tested showed signs of lysis (Table 3). Phage vB_EcoM_P5 was tested in *Enterobacteriaceae* culture collection strains (*n* = 10), patient isolates (*n* = 7), and other clinical isolates previously collected (*n* = 20). It lysed culture collection strains *E. coli* JM109 and *E. coli* MC1061, but none was lysed as efficiently as *E. coli* DSM 12242. No lytic activity was observed against the clinical isolates tested.

### 3.4. Antimicrobial Resistance Transduction

The antibiogram from *E. coli* isolate P5 revealed resistance to ampicillin (Table 2). Phage vB_EcoM_P5 isolated originally from this strain was used to test its ability to transduce ampicillin resistance. After 48h, no transductants were observed.

Given that the antibiogram *S. aureus* isolate P9 (Table 2) was sensitive to all the antibiotics tested, vB_SauS_P9 was first propagated in two resistant (clindamycin, penicillin G and erythromycin) isolates from surface sample P5, determined as high and medium producers (Table 3). Propagation in an AB-resistant bacterium may encourage phage-harboring resistance genes and thereby transduce antimicrobial resistance. After phage propagation in these strains, transduction was attempted using the sensitive *S. aureus* Sa9 as a recipient. No transductants were obtained.

### 3.5. Virucidal Effect of Disinfectants and Thermal Stability

**Figure 4:**
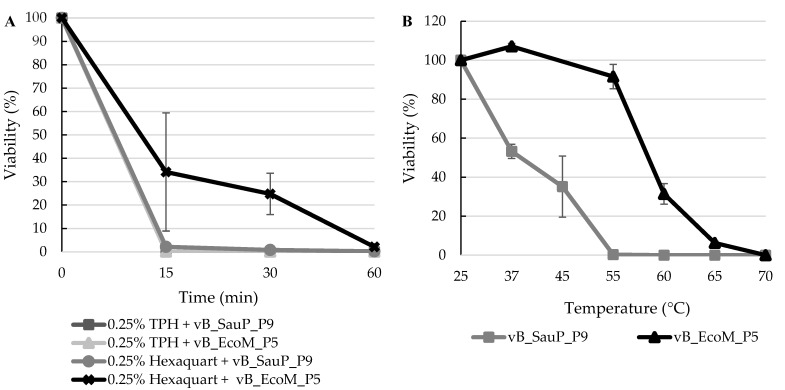
Stability of phages vB_EcoM_P5 and vB_SauS_P9 in the presence of disinfectants (**A**) and increasing temperatures (**B**).

The hospital disinfectant TPH Protect is widely used to disinfect the surfaces around patients at a concentration of 0.5% (v/v), according to hygiene measures applied in the facility. Half of this concentration was effective and inactivated vB_EcoM_P5 and vB_SauS_P9 after 15 min (Figure 4A). The coliphage was more stable to the disinfectant: vB_EcoM_P5 phage particles were still detected after 15 min (<0.1% survival), with total inactivation achieved after 30 min. The same highly virucidal effect was observed for vB_SauS_P9 in the presence of 0.25% Hexaquart® plus (2% survival after 15 min and 0.2% after 60 min). Coliphage vB_EcoM_P5 was better able to withstand the toxic conditions, displaying 34% survival after 15 min and 2% after 60 min. Both phages were still detected when using 0.5% Hexaquart® plus, but did not tolerate 70% ethanol. Phage vB_EcoM_P5 was generally more thermotolerant than staphylococci phage vB_SauS_P9 (Figure 4B), which is particularly evident at 55 °C, where phage counts were significantly reduced for vB_SauS_P9 (<1% survival), but vB_EcoM_P5 had a 92% viability. At 60 °C, all vB_SauS_P9 phage particles were inactivated. Phage vB_EcoM_P5 retained infectivity until 65 °C (6% survival), but was no longer detected at 70 °C.

## 4. Discussion

We present, to the best of our knowledge, the first study on the association between bacteriophages and bacteria in an ICU. Although *S. aureus* was found in the surroundings of all 12 patients and 47% of the samples were found to contain this species, no lytic phages were detected in any of the samples. The same result was obtained when testing for the presence of coliphages. Their apparent absence from clinical surfaces in the ICU might be associated with the lack of potential host *E. coli*, although it is possible that phages with a narrower host range remained undetected.

*S. aureus* is normally present in the flora of the human skin and is generally asymptomatic, although some strains are virulent and multidrug resistant [1,3]. Their occurrence is particularly problematic in an ICU, given the poor condition of patients—their increased vulnerability to infections and weakened immune systems [1,3]. A very high proportion (80%) of the *S. aureus* isolated showed multidrug resistance and these bacteria were found on surfaces within close proximity to patients. Frequently touched surfaces are highly contaminated by hospital staff and/or infected patients [7,41,42]. Objects in close contact to the patients, including textiles such as bed linen, pillows, mattresses, and pajamas, also represent vehicles for the carriage of hair-, skin- and gut-associated bacteria, such as *Staphylococcus* spp. [43,44]. The bacteria are extremely resistant to desiccation [41], which partly explains their success as a colonizer of the ICU. There is a clear risk of direct or indirect patient-to-patient transmission, which could potentially aggravate patients’ health. Antibiotic-resistant *Staphylococcus* sp. have been shown to be transmitted by aerosolization from bed linen during routine handling of bedding [43]. This possibility should be considered when identifying an appropriate disinfection routine, given the inherent resilience of staphylococci.

Despite the absence of lytic bacteriophages, two clinical isolates harbored mitomycin C-inducible prophages that were isolated and designated: vB_EcoM_P5 and vB_SauS_P9. These isolates came from urine obtained from a transurethral catheter and a blood culture. According to the 2016 Annual Epidemiological Report on healthcare-associated infections in ICUs in Europe, 97% of the reported cases of pneumonia was associated with intubation, with bloodstream and urinary tract infections also largely caused by catheters (43.6% and 99.3%, respectively) [6]. The presence of temperate phages associated with the bacteria might affect the outcome of the infection. Naturally occurring bacteriophages in the medical environment drive the fitness of bacterial pathogens. Mediating the horizontal transfer of virulence and antimicrobial resistance determinants can lead to more aggressive bacterial pathogens and hence a demand for more complex treatments [35,45,46]. Prophages at the onset of sepsis have a role in clonal selection during bacterial invasion [12]. On the other hand, phages have attracted interest as potential biocontrol agents [47,48,49], and bacteriophage-based products may be effective at eliminating or reducing the bacterial load in critical settings, such as hospitals [50]. Bacteriophage aerosols have been suggested as a possible alternative adjuvant to conventional surface disinfection routines in the ICU and their use decreased the rates of infection caused by carbapenem-resistant *Acinetobacter baumannii* [50]. One of the phages we isolated, *Staphylococcus* phage vB_SauS_P9, lysed the vast majority of the isolates collected from the surface samples of three patients colonized/infected with *Enterobacteriaceae* (P5, P7, P8), which might be clonally related and possibly dispersed in the ICU around patient 5. However, phage vB_SauS_P9 showed no activity against the clinical isolates tested.

We also investigated the possibility that these phages might act as vectors for horizontal gene transfer. Phages vB_EcoM_P5 and vB_SauS_P9 did not mediate the transfer of antimicrobial resistance from the selected donor bacteria to the sensitive host strains. The lack of appropriate selective pressure or genetic incompatibility of the mechanisms involved might have affected the generation of bacterial transductants. Despite their inability to transfer resistance determinants, the phages should not be regarded as safe for applications, given their temperate nature and possible integration in bacterial chromosomes [51].

We also tested the stability of phages vB_EcoM_P5 and vB_SauS_P9 to disinfectants. Staphylococci phage in the surgery suit of a horse clinic can persist after disinfection with Hexaquart® plus, which is based on a quarternary ammonium compound [52]. Such compounds have high efficiency against virulent dairy phages [53]. TPH Protect, the product used in the disinfection routine of the ICU we investigated, is a mixture of quaternary ammonium compounds and aromatic alcohol-based substances, with proven efficacy against enveloped viruses, *Rotavirus* and *Norovirus* [54]. Phage vB_EcoM_P5 was generally more thermotolerant and stable during disinfection than phage vB_SauS_P9. The phages were completely inactivated by 0.25% TPH Protect but could still be detected in 0.25–0.5% Hexaquart® plus. The phages were also unable to survive exposure to 70% ethanol, supporting the idea that a combination of alcohols and quaternary ammonium compounds can efficiently hinder viral activity. This practice, associated with the high frequency of disinfection in the ICU, might at least partly account for the absence of lytic phage particles in the surface samples.

Further investigation on the phages that inhabit the ICUs and their association with the bacteria living in this environment will help to understand phage ecology and critically assess phage applications in the future.

## Figures and Tables

**Table 1:** Type of sample, collection date, and number of sites analyzed for each patient (P1-P12).

Patient ID	Type of Sample	Collection Date	Number of Sites
P1	Gel swab	21.02.17	5
P2	Gel swab	21.02.17	5
P3	Gel swab	05.04.17	6
P4	Gel swab	11.05.17	7
P5 ^1^	Gel swab, liquid swab	21.06.17	6, 6
P6	Liquid swab	06.07.17	6
P7	Liquid swab	06.07.17	6
P8	Liquid swab	06.07.17	6
P9	Liquid swab	26.07.17	5
P10	Liquid swab	26.07.17	5
P11 ^2^	Surface wipe	26.03.18	6
P12 ^2^	Surface wipe	26.03.18	8

^1^ The sites analyzed in P5 were replicated to compare both sampling methods. ^2^ No isolates from the patient available.

**Table 2:** Clinical isolate, isolation source, and corresponding antibiogram.

Patient ID	Bacterial Isolate	Isolation Source	Colony Count ^2^	Antibiogram	Mitomycin C-Inducible Prophage
P1	*S. aureus*	Nose swab	+++	Sensitive	-
P2	*S. aureus*	Trachea secretion	+++	P ^R^, MXF ^R^	-
P3	*E. coli^1^*	Urine transurethral catheter	10^7^/mL	AM ^R^, SAM ^R^, CPD ^R^, CTX ^R^, CXM ^R^, CIP ^R^, CN ^R^, TMP ^R^	-
P4	*E. coli*	Urine transurethral catheter	10^4^/mL	AM ^R^, SAM ^R^	-
P5	*E. coli*	Urine transurethral catheter	10^7^/mL	AM ^R^, TMP ^R^	+
P6	*K. pneumoniae*	Trachea secretion	+++	AM ^R^	-
P7	*K. pneumoniae ^1^*	Trachea secretion	+	AM ^R^, SAM ^R^, CPD ^R^, CTX ^R^, CXM ^R^, TMP ^R^	-
P8	*K. pneumoniae*	Trachea secretion	+	AM ^R^	-
P9	*S. aureus*	Blood culture	Not detectable	Sensitive	+
P10	*K. oxytoca*	Trachea secretion	+	AM ^R^, SAM ^R^, CXM ^R^	-

^1^ ESBL (extended-spectrum β-lactamase), CRE isolate; ^R^ stands for resistant. ^2^ Colony counts were assessed either by direct counting or by indirect quantification using one “+” to “+++”.

**Table 3:** Antimicrobial activity of temperate phages vB_EcoM_P5 and vB_SauS_P9.

vB_EcoM_P5				vB_SauS_P9			
Bacteria	Lysis	E.O.P.	Reference and/or Source	Bacteria	Lysis	E.O.P.	Reference and/or Source
*Culture collection strains*				*Staphylococcus aureus from surface samples*			
*Escherichia coli* DSM 12242	+	1.000 ± 0.000	DSMZ	Surface P1 (0/9) ^1^	-	-	Environment, this study
*Escherichia coli* W3110 (ATCC 27325)	-	-	ATCC	Surface P2 (0/5)	-	-	Environment, this study
*Escherichia coli* JM109 (DSM 3423)	+	0.005 ± 0.001	DSM	Surface P3 (0/4)	-	-	Environment, this study
*Escherichia coli* DH5α (DSM 6897)	-	-	DSM	Surface P4 (0/2)	-	-	Environment, this study
*Escherichia coli* ATCC 11303	-	-	ATCC	Surface P5 (7/11)	+	0.142 ± 0.080–0.781 ± 0.219	Environment, this study
*Escherichia coli* MC1061 (ATCC 53338)	+	0.009 ± 0.001	ATCC	Surface P6 (0/8)	-	-	Environment, this study
*Klebsiella pneumoniae sub. pneumoniae* ATCC 13883	-	-	ATCC	Surface P7 (7/7)	+	0.000 ± 0.000–0.001 ± 0.000	Environment, this study
*Yersinia enterocolitica sub. palearctica* DSM11502	-	-	DSM	Surface P8 (7/11)	+	0.000 ± 0.000–0.013 ± 0.009	Environment, this study
*Salmonella enterica sub. enterica* ATCC 14028	-	-	ATCC	Surface P9 (0/5)	-	-	Environment, this study
*Salmonella typhimurium* DT104 isolate H3380	-	-	Human [40]	Surface P10 (0/7)	-	-	Environment, this study
*Patient isolates*				Surface P11 (0/1)	-	-	Environment, this study
*E. coli* isolate 19 (P3)	-	-	Human, this study	Surface P12 (0/3)	-	-	Environment, this study
*E. coli* isolate 33 (P4)	-	-	Human, this study	*Culture collection*			
*E. coli* isolate 46 (P5)	-	-	Human, this study	*Staphylococcus aureus* Sa9	+	1.0 ± 0.00	Food, [36]
*K. pneumoniae* isolate 77 (P6)	-	-	Human, this study	*Staphylococcus aureus* ATCC 33862	-	-	ATCC
*K. pneumoniae* isolate 76 (P7)	-	-	Human, this study	*Staphylococcus aureus* NCTC 6571	-	-	NCTC
*K. pneumoniae* isolate 75 (P8)	-	-	Human, this study	*Patient isolates*			
*K. oxytoca* isolate 89 (P10)	-	-	Human, this study	*S. aureus* isolate 11 (P1)	-	-	Human, this study
*Clinical isolates*				*S. aureus* isolate 12 (P2)	-	-	Human, this study
*E. coli* (*n* = 20)	-	-	Human, this study	*S. aureus* isolate 83 (P9)	-	-	Human, this study
				*Clinical isolates*			
				*S. aureus* isolate 24	+	0.017 ± 0.013	Human, this study
				*S. aureus* isolates (*n* = 16)	-	-	Human, this study

^1^ Number of isolates showing lysis/number of isolates tested.
